# Osmotically Regulated Two-Compartment Asymmetric Membrane Capsules for Simultaneous Controlled Release of Anti-Hypertensive Drugs

**DOI:** 10.3797/scipharm.1106-01

**Published:** 2011-12-23

**Authors:** Saurabh Garg, Kamla Pathak, Anil Philip, Dinesh Puri

**Affiliations:** 1 Amul Pharmaceutical Pvt. Ltd., Jaipur, Rajasthan, India; 2 Department of Pharmaceutics, Rajiv Academy of Pharmacy, Mathura, U. P., India; 3 Department of Pharmaceutics, School of Pharmacy, College of Pharmacy and Nursing, University of Nizwa, Birkat Al Mouz 616, Nizwa, Sultanate of Oman; 4 Faculty of Pharmaceutical Sciences, Jodhpur National University, Jodhpur, Rajasthan, India

**Keywords:** Atenolol, Amlodipine besylate, Asymmetric membrane capsules, Osmotically regulated

## Abstract

In the present study, asymmetric membrane capsules (AMCs) with two compartments were successfully developed for simultaneous delivery of two poorly water-soluble drugs, Atenolol and Amlodipine Besylate, by using solubility modulation approach. Scanning electron microscopy (SEM) before dissolution showed presence of outer dense region and inner porous region for the prepared asymmetric membrane and the pore size increased after dissolution for both outer and inner layer. Diffuse reflectance spectroscopy (DRS) showed no incompatibility between the drug(s) and the excipients used in the study. The developed system was able to control the release of ATN and AMB by increasing the solubility through buffering agents of different strengths (0.25N to 1.0N). As the level of buffering agent was increased, the solubility of drugs also increased inside the asymmetric membrane capsule. The developed system was independent of the agitation intensity of the dissolution fluid but was dependent on the polymer diffusibility and osmotic pressure of the media, which clearly stated that osmotic pumping was the primary mechanism of drug(s) release from AMCs. The results of *in-vitro* demonstration of effect of membrane thickness on dissolution fluid entering AMCs showed that as the membrane thickness increased the volume of dissolution fluid entering into AMC decreased. The release kinetic studies of different formulations of AMCs showed that formulation code six, which consists of the highest amount of osmotic agents and optimum amount of buffering agents, was the best formulation, and it followed zero order release kinetics (r^2^=0.9990 for ATN and r^2^=0.9988 for AMB).

## Introduction

Development of osmotically regulated multi-drug oral delivery system would have the possibility of simultaneous administration of two or more drugs for the treatment of chronic diseases such as hypertension. The system would provide simultaneous delivery of two or more drugs, which is required to reduce the problems associated with multi-drug therapy. In addition, the system may provide the release of drugs in a near zero order rate, which is an ideal release profile for controlled drug delivery that in turn would improve safety profile of the drugs and enhancement of activity duration for drugs exhibiting short half life. And, once-daily formulations (optimized therapy) would increase the improved patient compliance [1].

The asymmetric membrane capsule is a controlled drug delivery device which consists of a drug-containing core surrounded by a membrane which has an asymmetric structure, i.e., it has a relatively thin, dense region supported on a thicker, porous region. Similar to a conventional telescoping hard gelatin capsule, the asymmetric membrane capsule consists of a cap and a body that snugly fit into each other. The cap is shorter in length and has a slightly larger diameter than the body which is longer and has a smaller diameter.

Drug delivery from asymmetric membrane dosage forms is primarily controlled by the difference in osmotic pressure between the external fluid and drug-containing core of the dosage form. The mechanism of drug release from an AM capsule consists of imbibitions of water through the membrane into the tablet core, dissolution of soluble components (including drug) in the core and pumping of the solution out of pores in the membrane. The imbibitions of water through the membrane are driven by its thermodynamic activity gradient between the external medium, e.g., receptor solution or gastric/intestinal fluids, and the osmotic agent in the core. Dissolution of the soluble components within the core produces the activity gradient and establishes the osmotic pressure difference between the core and external environment.

As water diffuses into the core, the volume of the imbibed water creates a hydrostatic pressure difference across the membrane, which forces the solution out through the pores in the coating. Therefore, the rate of drug delivery will be constant as long as a constant osmotic pressure gradient is maintained across the membrane, the membrane permeability remains constant, and, the concentration of drug in the expelled solution is constant [2, 3].

Atenolol is a β-adrenergic receptor blocking agent without membrane stabilizing or intrinsic sympathomimetic activities, and Amlodipine Besylate is a calcium channel blocker. This combination is used for the treatment of hypertension. It is reported that in case of atenolol oral administration, the tablet is usually administered two or three times a day, which would lead to large fluctuation in drug plasma concentration and side effects like diarrhea, nausea, ischemic colitis and mesenteric arterial thrombosis on human body. Amlodipine Besylate is extensively metabolized by liver to inactive metabolites. Steady-state plasma levels of amlodipine besylate are reached after 7 to 8 days of consecutive daily dosing. This combination is commercially available as a conventional tablet. Both drugs have poor aqueous solubility, which would lead to large fluctuation in drug plasma concentration. Controlled release systems are highly desirable to solve these problems [4–6].

Therefore, the aim of this work was (1) to develop two-compartment asymmetric membrane capsules (AMC) for simultaneous controlled release of atenolol and amlodipine besylate and study the release of drugs from these formulations and (2) to evaluate the effect of membrane thickness on dissolution fluid entering the AMC and (3) to evaluate the effect of different osmotic pressure conditions, agitation intensity and polymer diffusibility on drug release from the prepared AMCs.

## Experimental

### Materials

Atenolol (ATN) was obtained as gift samples from IPCA Lab. Ltd., Ratlam, India and amlodipine besylate (AMB) was obtained as gift samples from Cadila pharmaceuticals, Ahmedabad, India. Sodium hydroxide, acetone, methanol and potassium chloride were procured from Qualigens Pvt. Ltd., Mumbai, India. Ehanol and potassium dihydrogen phosphate were procured from S.D. fine chemicals, Mumbai, India. Cellulose acetate, sorbitol 70% and citric acid monohydrate were procured from Fluka, U.K., Central drug house Ltd., New Delhi, India and Ranbaxy fine chemicals, New Delhi, India, respectively. All other chemicals used in study were of analytical grade.

### Methods

#### Drug-drug and drug-excipient(s) compatibility study

Drug-drug and drug-excipient(s) compatibility were carried out using diffuse reflectance spectroscopy (DRS). In this technique solid drug, excipient(s) and their physical mixtures were diluted with KBr (IR grade) to get the samples for measurement in the transmittance mode (%T). The diffuse reflectance spectrum of the samples against the diluting material was measured by setting the accumulation time to approximately 50. The spectra obtained were evaluated for any incompatibility.

#### Preparation of asymmetric membrane capsules (AMCs) of atenolol and amlodipine besylate

AMCs were produced by using a dip coating (wet phase inversion) process. The glass mold pins were dipped into polymer solution consisting of cellulose acetate (10 %w/v or 15%w/v) dissolved in a mixture of acetone, alcohol and sorbitol, followed by quenching in a 10% v/v aqueous solution of sorbitol for 10 min. After quenching, the pins were withdrawn and allowed to air dry. Then, the capsules were stripped off the pins, trimmed to size and kept into dessicator until use [3].

#### Formation of compartment, filling and sealing of AMCs

The physical mixture of Atenolol (50 mg) and osmotic agent KCl (25, 50 and 100 mg) and citric acid monohydrate (17.5 mg) were prepared by mixing them in polyethylene bag for at least 10 min and filled inside the capsule’s body, the physical mixture of Amlodipine Besylate (5 mg) and osmotic agent, KCl (5, 10 and 20 mg) and citric acid monohydrate (35 mg) were also prepared by mixing them in polyethylene bag for at least 10 min, and filled inside the capsule’s cap manually. Compartments were formed using paraffin wax plug (due to its inertness), and a layer of cellulose acetate solution (10% w/v) was applied over it to ensure non- leakage of drug solution from cap and body. After the filling operation, the capsules were capped and sealed with a sealing solution (10% w/v cellulose acetate in a mixture of acetone and alcohol).[Table t1-scipharm.2012.80.229]

### Characterization of AMCS of atenolol and amlodipine besylate

#### Appearance and dimension

The asymmetric membrane capsules were characterized for appearance and dimension. AMCs were compared visually with regard to transparency and opacity. Dimensions of AMCs were determined by using a vernier caliper. A multiple of three determinants was used for measurement of each dimension. The results of the studies were statistically compared with conventional hard gelatin capsules at P<0.05.

#### Scanning electron microscopy

Asymmetric membrane before and after completion of dissolution of core was examined for their porous structure and thickness using Leo 435 VF scanning electron microscope (SEM). After dissolution, asymmetric membrane structure was dried at 50°C for 8 hrs and stored in dessicator before examination. The asymmetric membrane was sputter coated for 5–10 min with gold by using fine coat ion sputter and examined under SEM.

#### In-vitro release studies

*In vitro* percent cumulative drug release from prepared formulations were studied by using USP paddle type apparatus with rotating speed 100 rpm and temperature set at 37±0.5 °C. The release media was 0.1 N HCl (pH 1.2) as simulated gastric fluid (SGF pH 1.2, 750 ml) for the first 2 h, followed by phosphate buffer as simulated intestinal fluid (SIF pH 7.4, 900 ml) for rest of experiment. Five ml of the sample was withdrawn at specified time intervals, and suitably diluted with fresh release media and analyzed at the drug(s) their respective λ_max_ 276 nm for Atenolole and 360 nm for Amlodipine wavelengths. The amount of cumulative percent released at each time point was calculated.

#### Kinetics of in-vitro release

In general the release of drug from an osmotic system depends on many factors such as osmotic pressure, pore size and coating thickness. The *in vitro* release from F_1_ formulation containing only drugs (50 mg atenolol and 5 mg amlodipine besylate, without the KBr and citric acid monohydrate) exhibited a limited drug release because of erratic dissolution profile at gastric pH therefore limited bioavailability 50–60% [7]. The release from the formulations containing osmotic agents and buffering agents was more controlled, with increased bioavailability 80–85%. The zero-order rate describes systems where drug release is independent of its concentration and is generally seen for poorly water-soluble drug in matrix, transdermals, etc [8].

Eq. 1 $${\text{Q}}_{\text{t}}={\text{k}}_{0}\text{t}$$

The first-order describes systems in which the release is dependent on its concentration (generally seen for water-soluble drugs in porous matrix) [9].

Eq. 2 $$\text{ln }{\text{Q}}_{\text{t}}=\text{ln }{\text{Q}}_{0}-{\text{k}}_{1}\text{t}$$

The Higuchi model describes the release of the drug from an insoluble matrix to be linearly related to the square root of time and is based on Fickian diffusion.

Eq. 3 $${\text{Q}}_{\text{t}}={\text{k}}_{\text{H}}{\text{t}}^{1/2}$$

The Hixson-Crowell cube root law describes the release of drug from systems where it depends on the change in surface area and diameter of the particles or tablets with time and mainly applies in the case of systems that dissolute or erodes over time.

Eq. 4 $${{\text{Q}}_{0}}^{1/3}-{{\text{Q}}_{\text{t}}}^{1/3}={\text{k}}_{\text{HC}}\text{t}$$

Where Q_t_ is the amount of drug released at time t, Q_0_ is the initial amount of the drug in the formulation, k_0_, k_1_, k_H_ and k_HC_ are release rate constants for zero order, first order, Higuchi model and Hixson-Crowell rate equations.

### Effect of variables on drug release

#### Effect of osmotic pressure on drug release

Osmotic pressure and effect of the osmotic agent inside the formulation plays a vital role in deciding the release of drug from asymmetric membrane capsules. To confirm the mechanism of drugs release, release studies of the optimized formulation were conducted in media of different osmotic pressure (11.08 mmHg, 27.68 mmHg, 55.32 mmHg and 110.64 mmHg). The reason for the selection of these osmotic pressures was to have hyper and hypo osmotic conditions, and study them in comparison to the selected asymmetric membrane capsules. This was to justify that osmotic pressure was the reason behind the drug release from these formulations.

To increase the osmotic pressure of the dissolution medium (SIF), potassium chloride (osmotically active solute) was added, and the pH was adjusted to 7.4±0.5. Release studies were performed in 900 ml of media using USP-2 (paddle type) dissolution apparatus (100 rpm). Five ml of the sample was withdrawn at specified time intervals and suitably diluted with fresh release media and analyzed at respective wavelengths to determine the amount of Atenolol and Amlodipine Besylate releases from each AMC [2].

#### Effect of agitation intensity

Release studies were carried out at three different speeds namely 50, 100 and 150 rpm using USP-2 apparatus at 37±0.5 °C, and their effects on release profile were studied by analyzing the amount of drugs released from the formulation at predetermined intervals at respective wavelengths and then comparing the profile by using one-way ANOVA [10].

#### Effect of polymer diffusibility

The diffusibility of drug molecules through the rate-controlling membrane of a polymer membrane permeation controlled drug delivery system from the optimized formulation was studied using both the formulation stored in a dessicator for 24 h and also that from a freshly fabricated drug delivery system device. In-vitro dissolution for 1 h was done with a sampling time of 10 min. One milliliter of the sample was withdrawn and suitably diluted and analyzed at respective wavelength. The effect of polymer diffusibility was calculated using equation 5 for AMCs that were freshly fabricated and equation 6 for those stored for 24 h.

Eq. 5 $${\text{D}}_{\text{p}}={{\text{H}}_{\text{p}}}^{2}/6{\text{t}}_{\text{l}}$$

Where D_p_ is the polymer diffusibility, H_p_ is the thickness of the polymer membrane, and t_l_ is the time axis intercept of the back extrapolation through the steady-state drug release data.

Eq. 6 $${\text{D}}_{\text{p}}={{\text{H}}_{\text{p}}}^{2}/3{\text{t}}_{\text{b}}$$

Where D_p_ is the polymer diffusibility, H_p_ is the thickness of the polymer membrane, and t_b_ is the negative time axis intercept of the back extrapolation through the steady-state drug release data [11].

### In-vitro demonstration of effect of membrane thickness on dissolution fluid entering the AMC

To demonstrate the effect of membrane thickness on dissolution fluid entering the AMC, the volume that enters in the capsules was determined. For the determination of volume, different osmotic pressures were created in the external media by adding different amounts of osmotic agent. It was assumed that when the osmotic pressure inside the capsule and external media was equal (i.e. iso-osmotic) there will be no release of drugs. The osmotic pressure of media, at which the release was zero, was determined and by the use of this osmotic pressure volume of dissolution fluid that entered into the AMC can be calculated by using following equation.

Eq. 7 πV=nRT

Where π is the osmotic pressure in atm, V is the volume of solution in liters, n is the number of moles of solute, R is the gas constant equal to 0.082 liter atm/mole deg, and T is the absolute temperature [12].

### Stability studies

Stability studies were carried out as per ICH Q_1_A stability guidelines. The formulated capsules were subjected to 40 °C±2.0 °C/75% RH±5% for 3 months, and the samples were evaluated for physical parameter and *in vitro* release by UV spectrophotometer at respective wavelengths. The sampling intervals were 0,1,2,3 months.

## Results and discussion

### Drug-Drug and drug-excipients compatibility studies

**Fig. 1:**
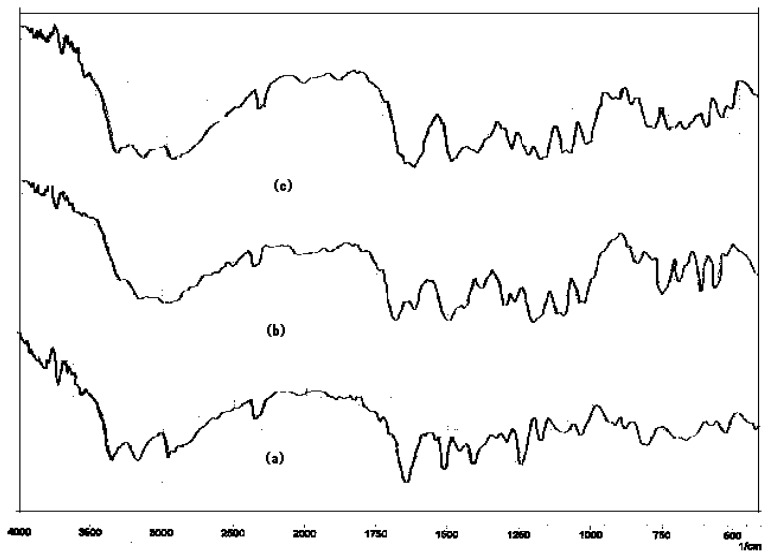
DRS spectra of (a) ATN, (b) AMB and (c) mixture of ATN and AMB

**Fig. 5:**
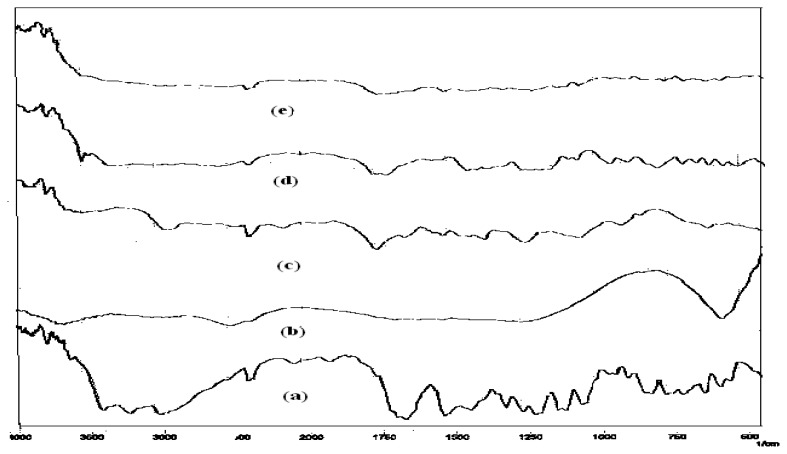
DRS spectra of (a) mixture of ATN and AMB, (b) KCl, (c) cellulose acetate, (d) citric acid monohydrate and mixture of ATN, AMB, KCl, CA and citric acid monohydrate

The physical mixture of drug and excipient(s) did not show any physical incompatibility in terms of discoloration, caking and liquefaction. The presence of excipient(s) did not result in any shift in the DRS of the drug(s) nor did it show the appearance of new peak (Fig. 1 to Fig. 5). DRS spectra of mixture of ATN and AMB along with polymers retained all the characteristic peaks of ATN and AMB and showed no incompatibility. Hence it can be concluded that AMCs prepared by cellulose acetate, KCl and citric acid monohydrate are stable in terms of physical and chemical stability.

### Characterization and evaluation of prepared capsules

The appearance and dimensions of asymmetric membrane capsules were studied. The appearance and dimensions of the AMCs were compared to conventional hard gelatin capsules (Table 2). AMCs were found to possess high opacity as compared to the conventional capsules. Comparison of the dimensions of the capsules showed that there is a high degree of similarity (P= 0.0012) in physical appearance between the conventional hard gelatin capsules (HGCs) and AMCs.

### Scanning electron microscopy (SEM)

**Fig. 6:**
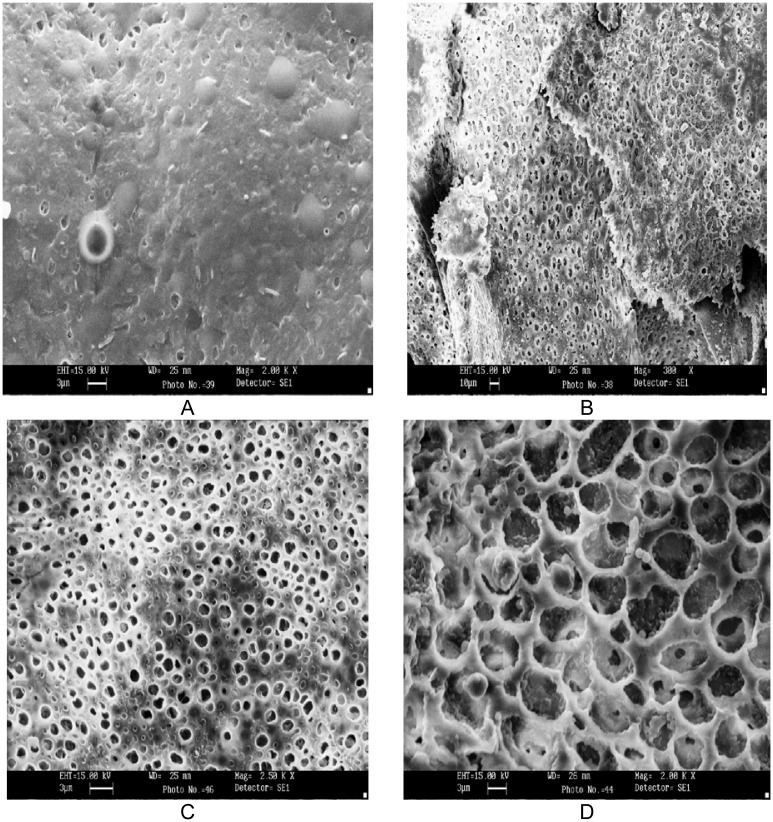
SEM of asymmetric membrane (A) outer dense membrane (B) inner porous region before dissolution and (C) outer dense region (D) inner porous region after complete dissolution, 10% w/w sorbitol

The photographs revealed the typical characteristics of asymmetric membrane with outer dense region and inner porous region (Fig. 6A and Fig. 6B). The original concept was to form an asymmetric membrane film consisting of a thick porous region to provide mechanical support and a thin dense region to provide perm selectivity. Scanning micrograph of asymmetric membrane confirmed that the asymmetric nature of the membrane is a function of plasticizer. The process followed for manufacturing of AMCs in lab was reproducible and produced asymmetric membranes structurally similar to the ones recorded in literature for osmotic drug delivery.

### In-vitro dissolution studies

The *in-vitro* dissolution studies were carried out in 0.1N HCl (pH 1.2) for the initial 2 h, and then followed by phosphate buffer (pH 7.4) for rest of time. The results of dissolution studied were compared by one-way ANOVA which was followed by Dunnett’s multiple comparison test in which each formulation was compared with a control formulation (F_1_) to test whether there was significant difference between different formulations compared to control formulation (F_1_). The result of ANOVA showed that F_cal_ value (5.296 for ATN and 7.15 for AMB) was more than F_tab_ value (2.34 for ATN and 2.39 for AMB) which was statistically significant at 95% confidence interval between the all six formulations. Then Dunnett’s multiple comparison test was applied to identify which formulation was different from control formulation (F_1_).

The result of Dunnett’s multiple comparison test showed that when F_2_ formulation, (which consisted of pure drugs and buffering agent) was compared with F_1_ formulation, which consisted of only pure drugs only, both dissolution profiles had statistically significant difference (q>2.574 & P<0.01) for both the drugs. F_1_ formulation showed less % cumulative release than F_2_ formulation, because both drugs were poorly water soluble so were unable to solubilize in dissolution media but by the incorporation of buffering agent solubility was increased, as in F_2_ formulation so % cumulative release was high in F_2_ formulation.

**Fig. 7:**
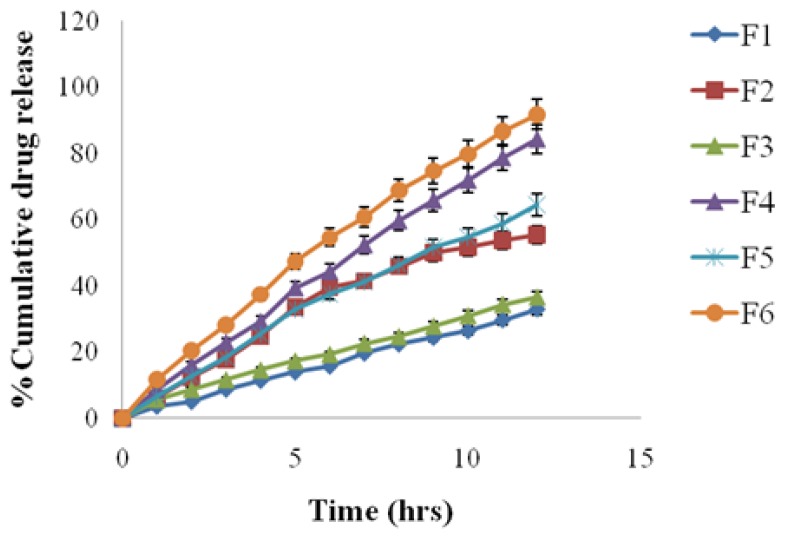
*In-vitro* drug release profiles of Atenolol in dissolution media

**Fig. 8:**
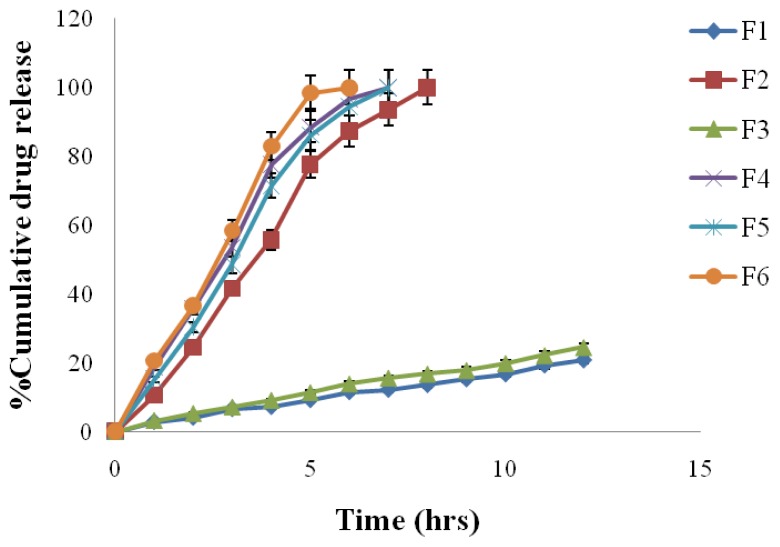
*In-vitro* drug release profiles of Amlodipine Besylate in dissolution media

When F_3_ formulation, which consisted of osmotic agent (KCl) along with pure drugs, was compared with F_1_ formulation both dissolution profiles had statistically insignificant difference (q<2.574 & P>0.05) for both drugs. F_3_ formulation showed slightly high % cumulative release than F_1_ formulation (Table 3, and Fig. 7., Fig. 8), because drug release due to solubility of drugs, but there maybe little contribution of the osmotic effect of osmotic agent for the drug that was solubilized in dissolution media.

When F_5_, F_4_ and F_6_ formulation, which consisted of low, medium and high amount of osmotic agent respectively and same amount of buffering agent along with pure drugs, were compared with F_1_ formulation individually, they were statistically significantly different (q>2.574 & P<0.01) from control formulation (F_1_) for both drugs. All three formulations (F4, F_5_ and F_6_) showed higher % cumulative release than F_1_ formulation (Table 3) due to the combined effect of both osmotic agent and buffering agent. Among the all three formulations (F_4_, F_5_ and F_6_), F_6_ formulation showed maximum % cumulative release because as the amount of osmotic agent increased, the % cumulative release also increased and release became more controlled.

### Modification of best formulation (F_6_)

**Fig. 9:**
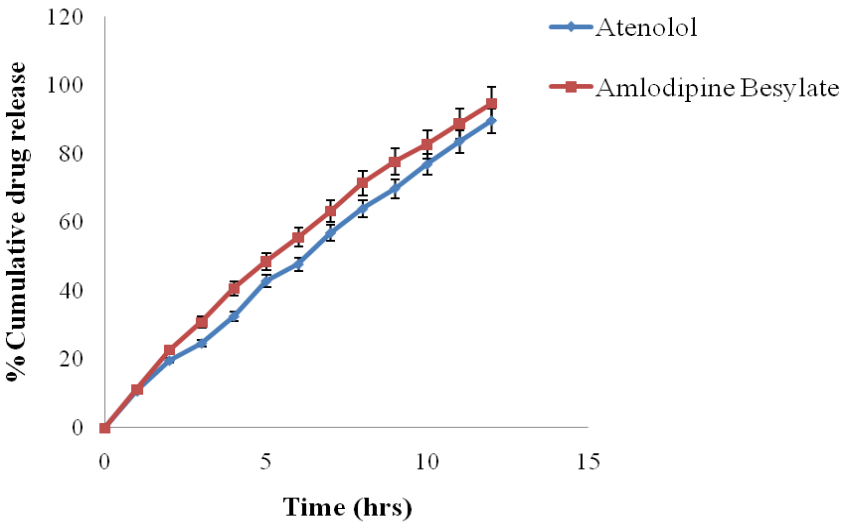
*In-Vitro* drug release profile of modified F_6_ formulation

The best formulation (F_6_) was modified because this formulation was not able to deliver Amlodipine Besylate up to 12 h, although it was delivering Atenolol up to 12 h. So the cap of the AMC was prepared with 15% w/v coating solution (cellulose acetate solution in acetone and alcohol) and the composition of body, which consisted of Atenolol, remained unchanged (i.e. 10% w/v cellulose acetate solution in acetone and alcohol). The result showed that after modification the formulation (F_6_) was able to deliver Amlodipine Besylate up to 12 h (Table 4 & Fig. 9).

### Kinetics of drug release

All the models for selecting the release profile were applied on all the AMC formulations (F_1_ to modified best formulation F′_6_). The results are summarized in Table 5. Results showed that best fit model in all the cases except F_2_ could have followed the Zero order, first order, Matrix model and the Peppas model. While considering higher correlation coefficient value (r^2^), the release data seems to fit Zero- order model better. According to correlation coefficient value (r^2^) of release models F_6_ seems to be the best formulation.

### Effect of variables on drug release

#### Effect of osmotic pressure on drug release

**Fig. 10:**
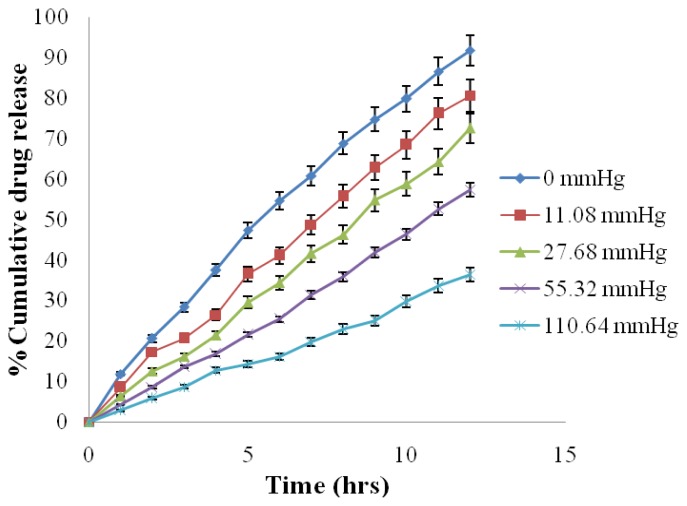
Effect of osmotic pressure on Atenolol release

**Fig. 11:**
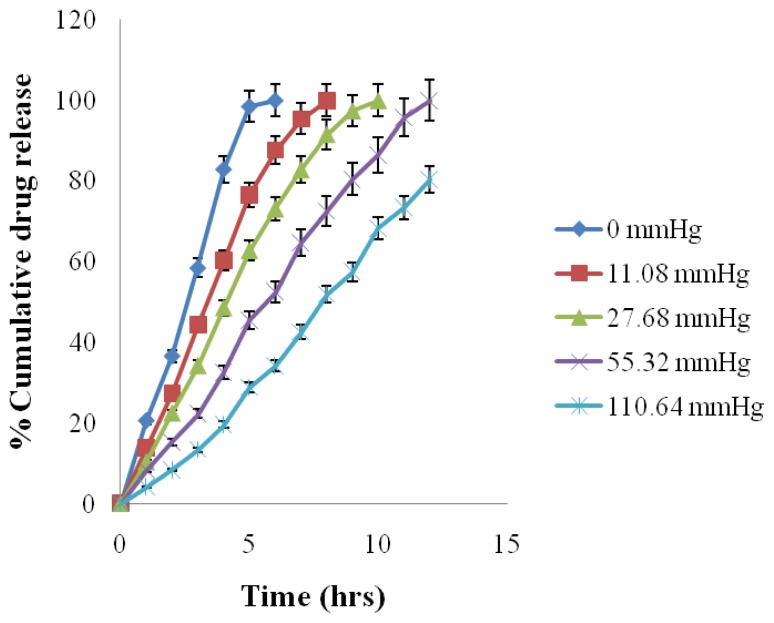
Effect of osmotic pressure on Amlodipine Besylate release

**Fig. 12:**
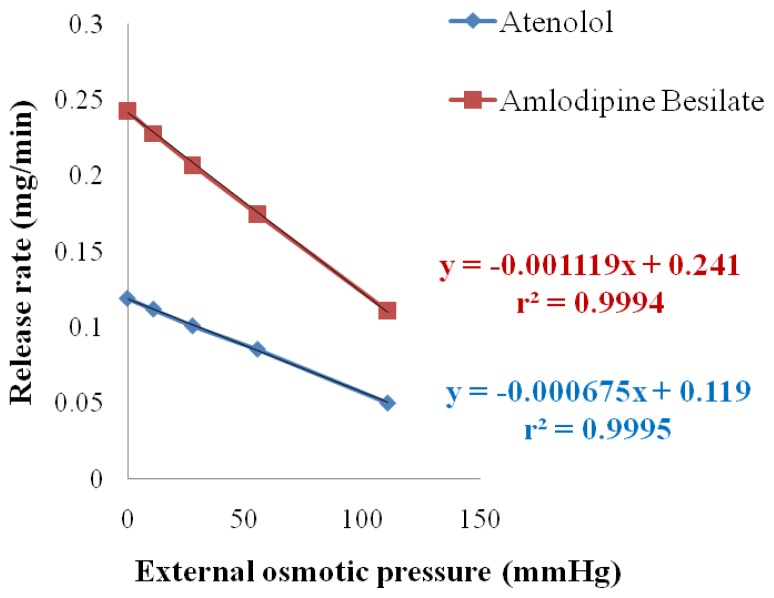
Demonstration of osmotic release from asymmetric membrane capsule

The result of release studies in media of different osmotic pressure showed that the *in vitro* release of ATN and AMB is highly dependent on the osmotic pressure of the release media (Table 6). Drug release from the formulation decreased as the osmotic pressure of the media increased (Fig. 10 & 11). On plotting graph between external osmotic pressure and release rate, the release rate decreases linearly with increase in external osmotic pressure (Fig. 12). It was concluded that osmotic pumping is the major mechanism governing the release from developed formulation [2].

### Effect of agitational intensity

The effect of agitational speed on the *in vitro* release of ATN and AMB was studied and the data is recorded in Table 7. Release studies of the best formulation (F_6_) were carried out in dissolution apparatus USP-2 at three different speeds i.e. 50rpm, 100rpm and 150rpm. Samples were withdrawn at predetermined intervals and analyzed by UV spectrophotometer. *In vitro* release was to found to follow the zero order release kinetics in all three cases determined by the PCP disso software. Release profile(s) at all three conditions were compared using one way ANOVA (Table 7). The calculated F value was found to be less than tabulated F value for both the drugs, thus suggesting that the variation in agitational intensity does not have any significant effect on release profiles of the asymmetric membrane tablets. This effect describes the fact that the *in-vitro* release from the AMCs is independent of the hydrodynamic conditions of the body [10].

### Effect of polymer diffusibility

**Fig. 13:**
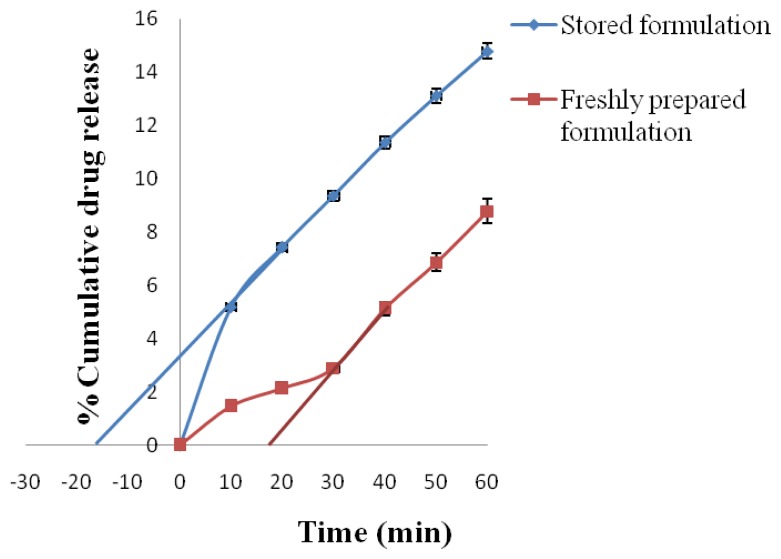
Effect of polymer diffusibility on ATN release

**Fig. 14:**
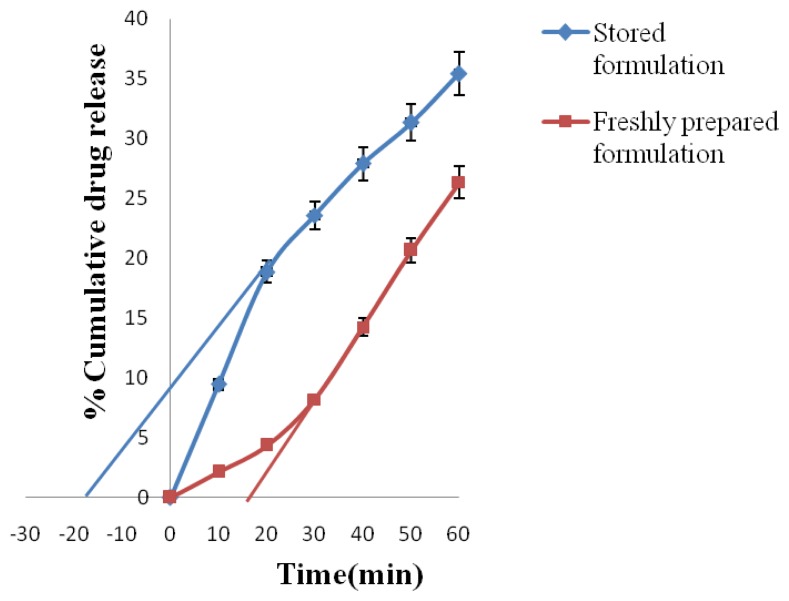
Effect of polymer diffusibility on AMB release

The effect of polymer diffusibility on drug release (since drug release results from diffusion of drug through asymmetric membrane barrier) from the best formulation (F_6_) was studied (Fig. 13& 14) using formulation that was stored in a dessicator for 24 h and also form a freshly fabricated drug delivery device. Polymer diffusibility from freshly prepared formulation was calculated to be 4959.84 μm/min and 5251.60 μm/min for ATN and AMB, respectively, and polymer diffusibility for formulation stored in a dessicator for 24 h was calculated to be −9397.60 μm/min and −8927.72 μm/min for ATN and AMB, respectively. A positive value for polymer diffusibility for the freshly prepared formulation suggests a lag time in release of ATN and AMB, which means that the drug has not penetrated the membrane (i.e. the drugs are not released until the dissolution medium has penetrated the membrane barrier) dissolving the drug in the reservoir, whereas a negative value for polymer diffusibility for the formulation stored for 24 h suggests saturation of ATN and AMB at the pores of the AMC. Because of this saturation of the drug at the pores of the membrane, when the dissolution medium enters the AMC the process of drug entering into the solution form will be faster, thereby resulting in faster release from the system. Polymer diffusibility studies suggest that the stored formulations may result in burst release before achieving steady state and can be an important parameter in determination of the minimum effective concentration required by the drugs [11].

The current time of 24 h was preferred based on our previous studies that the pore saturability for the asymmetric membrane capsules was attained within 24 h, and that the drug release profile from the formulations stored at 24 h or beyond were statistically similar.

Since the previous studies [11] have shown that a burst release is achieved within the first hour of release due to the pore saturability, the study was conducted for the same. Moreover, it was expected that the two formations (after the initial burst from the stored formulation) will have a similar drug release.[Table t8-scipharm.2012.80.229]

### In-vitro demonstration of effect of membrane thickness on dissolution fluid entering the AMC

To demonstrate the effect of membrane thickness on dissolution fluid entering the AMC, the volume that enters in the capsules was determined. For the determination of volume, different osmotic pressures (65.55 atm, 182.08 atm, 327.74 atm, 455.19 atm, 546.23 atm, 655.47 atm and 910.38 atm) were created in the external media by adding different amounts of osmotic agent (KCl). It was assumed that when the osmotic pressure inside the capsule and external media was equal (i.e. iso-osmotic) there will be no release of drugs. The osmotic pressure, at which the drug release was zero, was recorded for Atenolol and Amlodipine Besylate. The results showed that at 455.19 atm and 910.38 atm osmotic pressure Atenolol and Amlodipine Besylate release was found to be zero, respectively. Therefore, it was concluded that 455.19 atm and 910.38 atm osmotic pressure was developed inside the capsule’s body and cap, respectively, which was equivalent to external media osmotic pressure. The volume that was responsible for building up osmotic pressure inside the capsule was calculated for both compartments. The results showed that 0.072 ml fluid entered into the body of AMC and 0.0072 ml fluid entered into the cap of AMC, because in the modified F_6_ formulation cap of AMC was prepared by 15% w/v coating solution (cellulose acetate solution in acetone and alcohol) to achieve prolonged release of Amlodipine Besylate.[Table t9-scipharm.2012.80.229]

### Stability studies

The stability study was carried on with modified best formulation (F′_6_) according to ICH Q1 A guidelines for three months to investigate the influence of humidity and temperature on appearance and *in vitro* drug release. The results (table 10) revealed that the formulation was stable when store in sealed as well as unsealed containers at 40°C ± 2.0**/**75% RH ± 5 as. In terms of appearance the capsules texture and color remained unchanged, thus proving the stability of asymmetric membrane capsules. The capsules were also subjected to dissolution for determining the % drug release after 12 h and showed that formulations have comparable release profiles, thus suggesting that there was no problem of stability for asymmetric membrane tablets.

## Figures and Tables

**Fig. 2:**
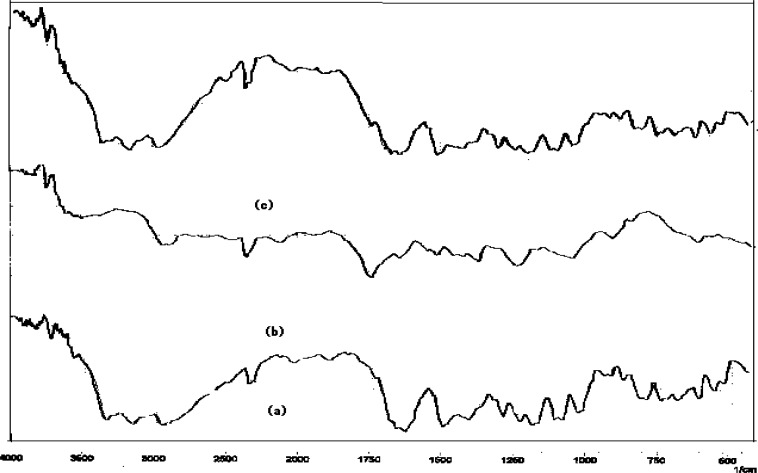
DRS spectra of (a) mixture of ATN and AMB, (b) cellulose acetate and (c) mixture of ATN, AMB and cellulose acetate

**Fig. 3:**
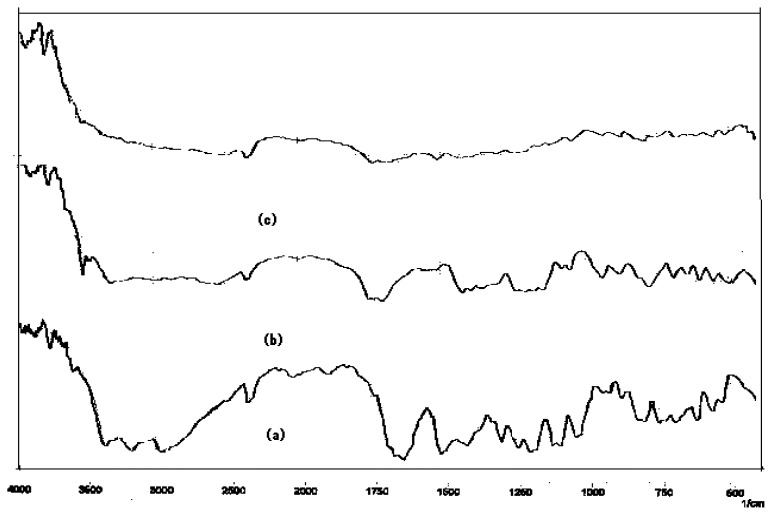
DRS spectra of (a) mixture of ATN and AMB, (b) citric acid monohydrate and (c) mixture of ATN, AMB and citric acid monohydrate

**Fig. 4:**
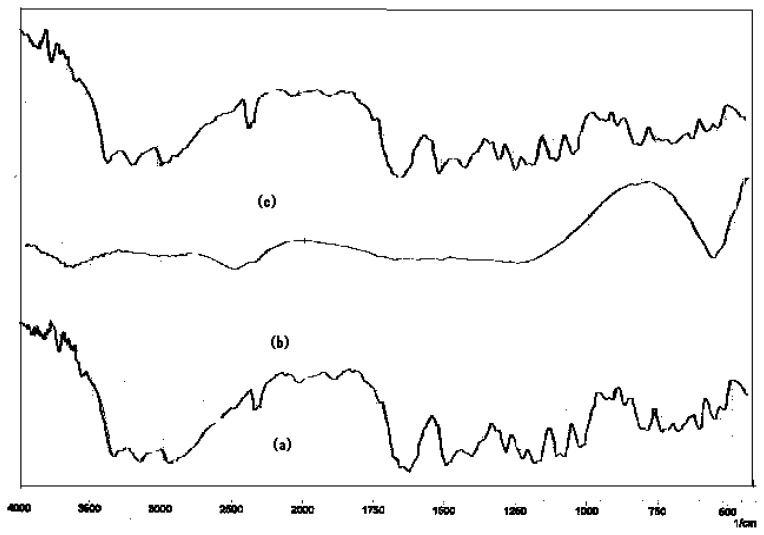
DRS spectra of (a) mixture of ATN and AMB, (b) KCl and (c) mixture of ATN, AMB and KCl

**Tab. 1 t1-scipharm.2012.80.229:** Formulation design of AMCs for simultaneous release of atenolol and amlodipine besylate

No.	COMPONENT	AMOUNT (mg)											
		
		F_1_		F_2_		F_3_		F_4_		F_5_		F_6_	
		
		C	B	C	B	C	B	C	B	C	B	C	B
1.	Atenolol	–	50	–	50	–	50	–	50	–	50	–	50
2.	Amlodipine Besylate	5	–	5	–	5	–	5	–	5	–	5	–
3.	Potassium chloride	–	–	–	–	10	50	10	50	5	25	20	100
4.	Citric acid monohydrate	–	–	35	17.5	–	–	35	17.5	35	17.5	35	17.5

C…Cap; B…Body.

**Tab. 2:** Physical characterization of AMC as compared to HGC

Type	Opaque	Size				

		Cap		Body		Sealed (mm)

		L (mm)	D (mm)	L (mm)	D (mm)	

HGC	+	10.12±0.11	7.85±0.12	18.02±0.13	7.02±0.12	21.85±0.11
AMC	++	10.21±0.14	7.95±0.13	18.23±0.14	8.12±0.13	22.43±0.23

**Tab. 3:** *In-vitro* release data of different formulations of ATN and AMB

Time (hr)	% Cumulative drug release											

	F_1_		F_2_		F_3_		F_4_		F_5_		F_6_	

	ATN	AMB	ATN	AMB	ATN	AMB	ATN	AMB	ATN	AMB	ATN	AMB
0	0	0	0	0	0	0	0	0	0	0	0	0
1	3.67±0.24	2.91±0.16	6.23±0.16	10.54±0.54	5.77±0.23	3.26±0.21	8.72±0.21	18.72±0.27	6.76±0.22	15.25±0.37	11.82±0.45	20.58±0.35
2	5.13±0.21	4.11±0.14	12.45±0.29	24.47±0.47	8.79±0.36	5.32±0.32	16.11±0.35	35.69±0.36	12.69±0.66	30.39±0.41	20.56±0.63	36.58±0.54
3	8.78±0.32	6.63±0.24	17.83±0.43	41.58±0.25	11.68±0.47	7.19±0.25	22.98±0.57	53.56±0.57	18.38±0.75	48.55±0.29	28.29±0.13	58.42±0.53
4	11.47±0.55	7.24±0.26	24.83±0.36	55.69±0.36	14.59±0.46	9.15±0.34	29.29±0.75	77.65±0.76	25.39±0.45	71.55±0.54	37.45±0.24	82.93±0.23
5	13.98±0.47	9.21±0.17	33.49±0.67	77.65±0.46	17.28±0.65	11.41±0.53	39.44±0.37	88.52±0.74	32.88±0.31	86.25±0.37	47.35±0.34	98.52±0.19
6	15.76±0.76	11.53±0.13	39.47±0.46	87.29±0.32	19.26±0.67	13.92±0.52	44.29±0.53	96.63±1.16	37.62±0.31	94.37±0.43	54.62±0.43	100±0.02
7	19.69±0.88	12.21±0.28	41.45±0.64	93.45±0.43	22.59±0.65	15.64±0.54	52.44±0.45	100±0.13	41.23±0.47	100±0.04	60.76±0.84	
8	22.43±0.56	13.59±0.32	45.76±0.76	100±0.08	24.62±0.38	16.83±0.37	59.63±0.63		46.33±0.56		68.81±0.75	
9	24.45±0.91	15.25±0.24	49.85±0.78		27.75±0.54	17.87±0.74	65.87±0.47		51.58±0.74		74.73±0.56	
10	26.44±0.45	16.68±0.43	51.52±0.56		30.92±0.37	19.92±0.62	71.92±0.39		54.69±0.47		79.83±0.57	
11	29.75±0.75	19.29±0.37	53.69±0.75		34.29±0.67	22.26±0.57	78.68±0.78		58.73±0.73		86.58±0.53	
12	32.98±1.24	20.75±0.23	55.37±0.64		36.5±0.78	24.59±0.51	84.29±0.87		64.46±0.57		91.76±0.54	

**Tab. 4:** *In-vitro* drug release data of modified formulation (F′_6_)

S.No.	Time (hrs)	% Cumulative drug release	

		Atenolol	Amlodipine Besylate
1.	0	0	0
2.	1	10.73±0.134	11.23±0.546
3.	2	19.68±0.154	22.76±0.647
4.	3	24.68±0.163	30.86±0.748
5.	4	32.56±1.420	40.75±0.843
6.	5	42.82±0.876	48.57±0.832
7.	6	47.76±0.433	55.57±0.174
8.	7	56.89±0.731	63.29±0.738
9.	8	63.92±0.674	71.52±0.736
10.	9	69.83±0.763	77.73±0.236
11.	10	76.83±0.772	82.73±1.312
12.	11	83.58±0.883	88.82±1.523
13.	12	89.63±0.912	94.69±0.646

**Tab. 5:** Different kinetic models applied on AMMC formulations

KINETIC MODEL	FORMULATION							

	F_1_		F_2_		F_3_		F_4_	

	ATN	AMB	ATN	AMB	ATN	AMB	ATN	AMB
Zero order (r^2^)	0.9867	0.9798	0.8979	0.8897	0.9944	0.9956	0.9980	0.9978
First order (r^2^)	0.9557	0.9628	0.9913	0.9924	0.9244	0.9239	0.8986	0.9014
Higuchi (r^2^)	0.9322	0.9230	0.8956	0.7857	0.9678	0.9668	0.9797	0.9762
Peppas (r^2^)	0.9719	0.9669	0.7845	0.7699	0.9953	0.9961	0.9962	0.9975

KINETIC MODEL	FORMULATION					

	F_5_		F_6_		F′_6_	

	ATN	AMB	ATN	AMB	ATN	AMB
Zero order (r^2^)	0.9979	0.9973	0.9990	0.9988	0.9985	0.9973
First order (r^2^)	0.8494	0.8776	0.8874	0.8644	0.8874	0.9632
Higuchi (r^2^)	0.9927	0.9841	0.9791	0.9792	0.9543	0.9543
Peppas (r^2^)	0.9934	0.9943	0.9937	0.9938	0.9949	0.9970

**Tab. 6:** Effect of varying external osmotic pressure on *in-vitro* release

Time (hr)	% Cumulative drug release									

	0 mmHg		11.08 mmHg		27.68 mmHg		55.32 mmHg		110.64 mmHg	

	ATN	AMB	ATN	AMB	ATN	AMB	ATN	AMB	ATN	AMB
0	0	0	0	0	0	0	0	0	0	0
1	11.82±0.23	20.58±0.33	8.52±0.13	13.76±0.19	6.39±0.62	10.37±0.77	4.32±0.21	8.35±0.13	2.78±0.43	4.25±0.11
2	20.56±0.32	36.58±0.54	17.28±0.32	27.29±0.76	12.53±0.43	22.45±0.98	8.57±0.23	15.27±0.77	5.81±0.25	8.38±0.16
3	28.29±0.45	58.42±0.11	20.63±0.59	44.39±0.59	16.12±0.53	34.25±1.09	13.56±0.66	22.35±0.63	8.63±0.53	13.42±0.34
4	37.45±0.22	82.93±0.55	26.43±0.52	60.29±0.24	21.39±0.53	48.57±0.65	16.76±0.42	32.56±0.52	12.76±0.88	19.62±0.55
5	47.35±0.25	98.52±0.32	36.58±0.66	76.62±0.22	29.46±0.55	62.77±0.88	21.53±0.53	45.51±0.25	14.29±0.53	28.83±0.24
6	54.62±0.31	100±0.17	41.12±0.74	87.67±0.23	34.42±0.58	73.12±0.43	25.35±0.61	52.58±0.27	16.12±0.27	34.23±0.27
7	60.76±0.22		48.74±0.77	95.41±0.17	41.58±0.34	82.77±0.56	31.39±0.87	64.79±0.51	19.76±0.73	42.58±0.31
8	68.81±0.15		55.71±1.22	100±0.08	46.23±0.75	91.56±0.55	35.83±0.33	72.58±0.42	22.93±0.53	51.87±0.42
9	74.73±0.25		62.78±0.11		54.79±0.63	97.43±0.34	41.78±0.19	80.44±0.87	24.92±0.25	57.48±0.43
10	79.83±0.47		68.45±0.25		58.77±0.63	100±0.55	46.38±0.57	86.46±0.44	29.78±0.22	68.33±0.47
11	86.58±0.55		76.17±0.44		64.24±0.55		52.58±0.54	95.76±0.27	33.65±0.36	73.44±0.63
12	91.76±0.44		80.58±0.34		72.51±0.75		57.39±0.71	100±0.16	36.38±0.66	80.44±0.14

**Tab. 7:** *In-vitro* release data for studying the effect of agitation intensity

Time (hr)	% Cumulative drug release					

	50 rpm		100 rpm		150 rpm	

	ATN	AMB	ATN	AMB	ATN	AMB
0	0	0	0	0	0	0
1	9.78±0.121	18.57±0.123	11.82±0.142	20.58±0.152	11.29±0.172	21.25±0.123
2	18.52±0.132	34.29±0.132	20.56±0.147	36.58±0.157	18.23±0.162	35.28±0.142
3	21.62±0.132	57.25±0.141	28.29±0.341	58.42±0.172	26.45±0.217	60.49±0.163
4	31.57±0.142	80.27±0.162	37.45±0.231	82.93±0.163	34.69±0.160	85.92±0.173
5	40.36±0.142	95.57±0.152	47.35±0.211	98.52±0.129	45.57±0.163	98.31±0.183
6	46.72±0.135	100±0.0120	54.62±0.238	100±0.0110	48.85±0.182	100±0.018
7	56.12±0.152		60.76±0.182		58.65±0.173	
8	60.24±0.141		68.81±0.178		65.86±0.172	
9	67.77±0.153		74.73±0.175		71.23±0.152	
10	73.98±0.152		79.83±0.163		78.47±0.183	
11	80.74±0.154		86.58±0.173		85.57±0.256	
12	87.36±0.172		91.76±0.193		90.5±0.162	

**Tab. 8 t8-scipharm.2012.80.229:** *In-vitro* release data for studying the effect of polymer diffusibility on ATN and AMB release

S.No.	Time (min)	% Cumulative drug release			

		Freshly prepared formulation		Stored formulation	

		ATN	AMB	ATN	AMB
1.	0	0	0	0	0
2.	10	1.45±0.110	2.12±0.115	5.19±0.113	9.46±0.135
3.	20	2.12±0.114	4.35±0.117	7.42±0.115	18.87±0.144
4.	30	2.89±0.118	8.22±0.171	9.37±0.212	23.58±0.121
5.	40	5.12±0.121	14.24±0.129	11.36±0.141	27.89±0.110
6.	50	6.87±0.151	20.67±0.153	13.11±0.143	31.34±0.171
7.	60	8.79±0.119	26.34±0.211	14.78±0.311	35.43±.211

**Tab. 9 t9-scipharm.2012.80.229:** *In-vitro* release data for demonstration of effect of membrane thickness on dissolution fluid entering the AMC

S. No.	Time (min.)	% Cumulative drug release							

		65.55 atm		182.08 atm		327.74 atm		455.19 atm	

		ATN	AMB	ATN	AMB	ATN	AMB	ATN	AMB
1.	0	0	0	0	0	0	0	0	0
2.	30	0.69	1.17	0.56	0.87	0.34	0.65	0	0.4
3.	60	1.56	2.58	1.29	2.05	0.72	1.21	0	0.78
4.	90	2.43	3.7	2.09	3.28	1.06	1.71	0	1.07
5.	120	3.39	5.18	2.89	4.23	1.41	2.31	0	1.55

S. No.	Time (min.)	% Cumulative drug release					

		546.23 atm		655.47 atm		910.38 atm	

		ATN	AMB	ATN	AMB	ATN	AMB
1.	0	0	0	0	0	0	0
2.	30	0	0.35	0	0.25	0	0
3.	60	0	0.64	0	0.52	0	0
4.	90	0	0.96	0	0.79	0	0
5.	120	0	1.31	0	1.04	0	0

**Tab. 10:** Compiled data for stability testing of asymmetric membrane capsules of ATN and AMB

Time interval (months)	Parameters		

	Appearance	Maximum % *in-vitro* release (after 12 h)	

		ATN	AMB
0	White & smooth	89.63	94.69
1	White & smooth	89.56	94.47
2	White & smooth	88.78	93.44
3	White & smooth	88.59	93.27
